# Longitudinal changes in plasma hemopexin and alpha-1-microglobulin concentrations in women with and without clinical risk factors for pre-eclampsia

**DOI:** 10.1371/journal.pone.0226520

**Published:** 2019-12-16

**Authors:** Katja Murtoniemi, Grigorios Kalapotharakos, Tero Vahlberg, Katri Räikkonen, Eero Kajantie, Esa Hämäläinen, Bo Åkerström, Pia M. Villa, Stefan R. Hansson, Hannele Laivuori

**Affiliations:** 1 Medical and Clinical Genetics, University of Helsinki and Helsinki University Hospital, University of Helsinki, Finland; 2 Department of Obstetrics and Gynaecology, University of Turku and Turku University Hospital, Turku, Finland; 3 Skåne University Hospital, Department of Clinical Sciences Lund, Department of Obstetrics and Gynecology, Lund University, Lund, Sweden; 4 Department of Clinical Medicine, Biostatistics, University of Turku and Turku University Hospital, Turku, Finland; 5 Department of Psychology and Logopedics, Faculty of Medicine, University of Helsinki, Helsinki, Finland; 6 PEDEGO Research Unit, MRC Oulu, Oulu University Hospital and University of Oulu, Oulu, Finland; 7 National Institute for Health and Welfare, Helsinki, Finland; 8 Children`s Hospital, University of Helsinki and Helsinki University Hospital, Helsinki, Finland; 9 Department of Clinical and Molecular Medicine, Norwegian University of Science and Technology, Trondheim, Norway; 10 Department of Clinical Chemistry, University of Helsinki, Helsinki, Finland; 11 Division of Infection Medicine, Department of Clinical Sciences, Lund University, Lund, Sweden; 12 Department of Obstetrics and Gynecology, University of Helsinki and Helsinki University Hospital, Helsinki, Finland; 13 Institute for Molecular Medicine Finland, Helsinki Institute of Life Science, University of Helsinki, Helsinki, Finland; 14 Department of Obstetrics and Gynecology, Tampere University Hospital and Tampere University, Faculty of Medicine and Health Technology, Tampere, Finland; University of Mississippi Medical Center, UNITED STATES

## Abstract

Recent studies have shown increased concentration of fetal hemoglobin (HbF) in pre-eclamptic women. Plasma hemopexin (Hpx) and alpha-1-microglobulin (A1M) are hemoglobin scavenger proteins that protect against toxic effects of free heme released in the hemoglobin degradation process. We used an enzyme-linked immunosorbent assay to analyze maternal plasma Hpx and A1M concentrations at 12–14, 18–20 and 26–28 weeks of gestation in three groups: 1) 51 women with a low risk for pre-eclampsia (LRW), 2) 49 women with a high risk for pre-eclampsia (PE) who did not develop PE (HRW) and 3) 42 women with a high risk for PE who developed PE (HRPE). The study had three aims: 1) to investigate whether longitudinal differences exist between study groups, 2) to examine if Hpx and A1M concentrations develop differently in pre-eclamptic women with small for gestational age (SGA) fetuses vs. pre-eclamptic women with appropriate for gestational age fetuses, and 3) to examine if longitudinal Hpx and A1M profiles differ by PE subtype (early-onset vs. late-onset and severe vs. non-severe PE). Repeated measures analysis of variance was used to analyze differences in Hpx and A1M concentrations between the groups. We found that the differences in longitudinal plasma Hpx and A1M concentrations in HRW compared to HRPE and to LRW may be associated with reduced risk of PE regardless of clinical risk factors. In women who developed PE, a high A1M concentration from midgestation to late second trimester was associated with SGA. There were no differences in longitudinal Hpx and A1M concentrations from first to late second trimester in high-risk women who developed early-onset or. late-onset PE or in women who developed severe or. non-severe PE.

## Introduction

Pre-eclampsia (PE) is a hypertensive disease that occurs in 2–8% of pregnancies. Globally, PE is a major cause of maternal and fetal morbidity and mortality [1, 2] and it indicates an elevated risk for subsequent long-term, non-communicable diseases of the mother and the newborn child [3, 4]. The exact cause of PE is unknown, but the understanding of the pathophysiological mechanisms of the disease has increased gradually. Currently, most popular theory is that the disease develops in two stages. The first stage occurs during early placental development, when impaired invasion of extravillous trophoblasts to the maternal spiral arteries causes defective remodeling of the arteries and incomplete vascular adaptation to pregnancy [5]. This causes uneven blood flow in the intervillous space of the placenta [6], intermittent hypoxia and oxidative stress [7]. Damages to the placenta-blood barrier follows and there is increased leakage of placental and fetal products into the maternal blood circulation [8–10]. It is known that poor placentation is associated with early-onset PE and small for gestational age (SGA) neonates, which may or may not be associated with PE [11].

The second stage of PE occurs later in pregnancy, after 20 weeks of gestation (GW) and the typical clinical manifestations are hypertension and proteinuria. This stage is characterized by endothelial dysfunction [12], maternal hypovolemia, vasoconstriction [13] and inflammation [14]. Circulating toxic factors derived from the placenta trigger an inflammatory response and induce general endothelial dysfunction. General organ damage develops, which, in turn, leads to the typical clinical manifestations of PE. Maternal susceptibility and certain maternal characteristics (e.g., obesity, metabolic syndrome, hypertension) play an important role at this stage [11, 15].

Earlier studies suggest that the plasma concentrations of free fetal hemoglobin (HbF) are increased in women who will develop PE and that HbF plays an important role bridging stage I and II [16–19]. The production of HbF is increased in PE placentas [20]. Extracellular HbF induces oxidative stress by forming reactive oxygen species (ROS). This, in turn, may result in damage to the placenta-blood barrier and leakage of extracellular HbF into the maternal circulation. Circulating extracellular hemoglobin (Hb) itself is a potent toxin, and free heme released during the degradation process of Hb exerts also oxidative and toxic effects on surrounding cells and tissue [21]. In fact, high levels of cell-free HbF in the umbilical cord correlate with fetal growth restriction (FGR) [22].

Hemopexin (Hpx) and alpha-1-microglobulin (A1M) are proteins involved in the hemoglobin scavenger system that protects against the toxic effects of free heme released during Hb degradation. Haptoglobin binds Hb and is the primary protective scavenger protein. Hemopexin has high affinity to free heme and is regarded as a back-up system when the Hb-binding capacity of haptoglobin is exceeded [23], although it does function independently of haptoglobin and does not require total haptoglobin depletion [24]. Plasma Hpx directs heme primarily to the parenchymal cells of the liver for catabolism, iron storage and redistribution; by binding free heme Hpx protects the endothelium [25]. Hpx has also enzymatic activity which increases during pregnancy and may be lead to reduced angiotensin II sensitivity during normal pregnancy [26]. A1M has heme-binding and heme-degrading [27] as well as enzymatic reductase properties which counteract heme-induced and ROS-induced cellular oxidation [28]. Both Hpx and A1M are potential biomarkers for predicting PE [19, 29] and several reports suggest that A1M may be a therapeutic target for PE [30–32], the most recent one for the mice PE model Storkhead box 1 (STOX-1) [33].

There are studies on plasma Hpx and A1M concentrations during the first trimester of pregnancy [19] and after manifestation of PE in late pregnancy [29]. In a previous study on maternal plasma Hpx and A1M concentrations in late second trimester we found that, during 26–28 GW, women at high risk for PE who did not develop PE had higher A1M concentrations than women at high risk who did develop PE [34]. In the present study we followed the plasma concentrations of these hemoglobin scavenger proteins at three time points during pregnancy in three different groups: women with a low risk for PE, in women with a high risk for PE who did not develop PE and in women with a high risk for PE who did develop PE. The focus in this study is on the longitudinal changes of Hpx and A1M concentrations during pregnancy. The aim was to clarify whether there are differences in Hpx and A1M concentrations longitudinally between the study groups and to increase our understanding of the pathophysiology of PE.

A recent study showed that the toxicity of HbF causes pathophysiological processes which lead to a compromised feto-placental circulation and FGR [22]. Since HbF is toxic to the fetus, the role of Hpx and A1M was also evaluated in women with PE who experienced FGR complications and who did not experience FGR complications. For this, we studied pre-eclamptic women with an SGA fetus (PESGA) and high-risk women who developed PE and who delivered an appropriate for gestational age (AGA) neonate (PEAGA).

Our third aim was to investigate if there are differences in longitudinal Hpx and A1M concentrations depending on the subtype of the PE, i.e., between early-onset PE vs. late-onset PE and between non-severe vs. severe PE.

## Materials and methods

This nested case-control study is a part of the multidisciplinary *Prediction and Prevention of Pre-eclampsia and Intrauterine Growth Restriction* (PREDO) project [35]. Women with known risk factors for PE were prospectively recruited between September 2005 and June 2009 at ten maternity clinics in Finland. The ethics Committee of the Helsinki and Uusimaa Hospital District approved the study and written informed consent was obtained from all participants.

**Fig 1:**
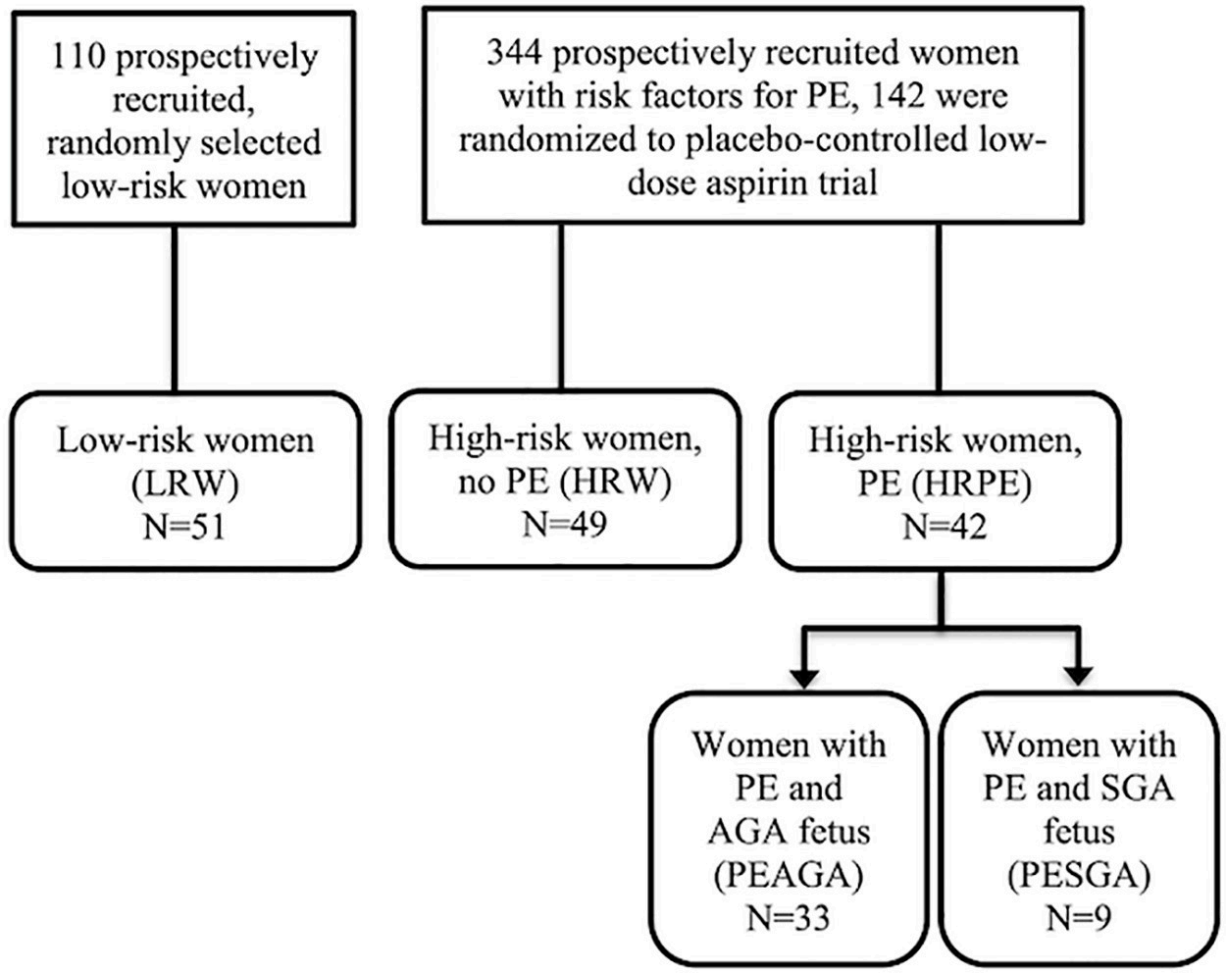
Flowchart of study cohort. PE = pre-eclampsia, SGA = small for gestational age, AGA = appropriate for gestational age.

In total 142 women were included: 42 women with PE (HRPE), 49 randomly selected high-risk women who did not develop PE (HRW) and 51 low-risk women (LRW). Seven women in the HRPE participated also in the acetylsalicylic acid (ASA) trial arm of the PREDO project and were treated with a daily low oral dose of acetylsalicylic acid (LDA 100 mg/d) starting before 14^th^ GW. The inclusion and exclusion criteria are described in Table 1 and the flowchart in Fig 1.

**Table 1:** Inclusion and exclusion criteria for high-risk women.

**Inclusion criteria**
Obesity (body mass index over 30 kg/m^2^)
Chronic hypertension (≥140/90 mmHg or antihypertensive medication before GW 20)
Sjögren’s syndrome
History of gestational diabetes
History of pre-eclampsia (blood pressure ≥140 mmHg systolic or ≥90 mmHg diastolic and proteinuria ≥0.3 g/day or dipstick equivalent in two consecutive measurements)
History of small for gestational age (birthweight < -2SD)
History of fetus mortus (fetal death after 22 weeks of gestation or >500 g weight in a previous pregnancy)
Systemic lupus erythematosus Type 1 diabetes mellitus
**Exclusion criteria**
Tobacco smoking (during this pregnancy)
Multiple pregnancy
History of asthma
History of peptic ulcer
Placental ablation
Inflammatory bowel diseases (Crohn’s disease, ulcerative colitis)
Rheumatoid arthritis
Hemophilia or thrombophilia (previous venous or pulmonary thrombosis or coagulopathy)

The criteria for PE were systolic blood pressure ≥ 140 mmHg and/or a diastolic blood pressure ≥ 90 mmHg after 20 GW and a urinary 24-h protein excretion of ≥ 0.3 g or dipstick equivalent in two consecutive measurements. Pre-eclampsia superimposed on chronic hypertension were included in the primary outcome. Secondary outcomes were PE combined with SGA infant, early-onset pre-eclampsia (diagnosed before 34^+0^ weeks of gestation) and severe pre-eclampsia (systolic blood pressure ≥ 160 mmHg and/or diastolic blood pressure ≥ 110 mmHg and/or proteinuria ≥ 5 g/24 hours). SGA was defined as a birthweight ≤ - 2 SDs [36].

All participants had their first visit at 12–14 GW. Uterine artery blood flow was measured with Doppler sonography. Gestational age was confirmed by crown-rump length measurement. The first-trimester mean arterial pressure (MAP) was calculated with the equation: MAP = diastolic blood pressure + (systolic blood pressure–diastolic blood pressure)/3.

The plasma Hpx concentration was measured using a sandwich ELISA kit (Human Hemopexin, Genway Biotech Inc., San Diego, CA, USA) and a multilabel counter (Wallac 1420, Perkin-Elmer, Wallac, Turku, Finland) (absorbance at 450nm).

Plasma A1M concentration was measured using an in-house ELISA and microtiter plates, which were coated with mouse monoclonal anti-A1M antibodies (clone 35.14). The monoclonal anti-A1M antibodies were produced against human urinary A1M by Agrisera AB (Vännäs, Sweden). Human urinary A1M was prepared in the laboratory of the Section for Infection Medicine, Department of Clinical Sciences, Lund University, Lund, Sweden, as described earlier [37].

Briefly, the plasma samples were incubated overnight at +4°C under sealing film in 5 μg/ml solution of phosphate buffered saline (PBS) buffer (+ 0.05% tween-20, 100 μl/well). After washing three times with PBS buffer, 100 μl of human urinary A1M reference standard (1.56–100 ng/ml in PBS buffer) or unknown diluted plasma samples (1:1000 with PBS buffer) were added to the wells and incubated for 1 h at room temperature, in darkness and on a rotational shaker (250–500 rpm). After washing three times with PBS buffer, 100 μl of the detection antibody solution was added (horseradish peroxidase-coupled mouse monoclonal anti-A1M antibody clone 57.10; in PBS) and incubated for 1 h at room temperature, in darkness and on a rotational shaker (250–300 rpm). After washing three times with PBS, 100 μl of 3,3',5,5'-tetramethylbenzidine (TMB substrate, SureBlue^TM^ TMB Microwell Peroxidase Substrate, KPL cat. no. 50-00-04) was added, sealed and again incubated for 20 min without shaking, and the reaction was stopped by adding 100 μl of 1 M sulfuric acid. Absorbance at 450 nm was read using the same Wallac 1420 Multilabel Counter.

### Statistical analyses

Data was analyzed for normality. The differences of means of normally distributed baseline variables between the groups were analyzed by one-way analysis of variance (ANOVA) followed by Tukey’s post-hoc tests. The Kruskal-Wallis test was used if the data was not normally distributed. Comparisons of medians of non-normally distributed variables were made with Mann-Whitney’s U test. Bonferroni corrections were used in post-hoc comparisons. Categorical variables were compared with Fisher’s exact test and in pairwise comparisons Bonferroni correction was used.

Repeated measures analysis of variance (RMANOVA) was used to analyze differences in plasma Hpx and A1M concentrations between the groups. The model included the main effects of time, group and the interaction effect time x group. Log-transformed Hpx and A1M values were used in these analyses due to positively skewed distributions. The best covariance structure was selected by using AICC (Akaike information criterion with a correction for small sample sizes) and compound symmetry covariance structure was chosen. The results of the values for Hpx and A1M are expressed as geometric means with 95% confidence intervals. One-way ANOVA followed by Tukey’s post-hoc tests was used for multiple comparisons of biomarker concentrations at each sampling point, if further analyses were indicated after RMANOVA. In all analyses a p-value < 0.05 was considered statistically significant. Statistical analyses were performed using version 25.0 of the SPSS statistic software package.

## Results

### Baseline characteristics

The baseline characteristics of LRW, HRW and HRPE are shown in Table 2 including the division of HRPE into two groups: 1. PESGA and 2. PEAGA. One woman in the HRW group had type 1 diabetes mellitus and one had Sjögren´s syndrome (Table 2). HRW and HRPE were older (p = 0.007) and had a higher body mass index (BMI) (p<0.001) than LRW. There were more women with chronic disease in the HRW and HRPE groups than in in the LRW group (p<0.001). Due to the inclusion criteria of the study there were, in the HRW and HRPE groups compared to the LRW group, less primiparous women (p<0.001) and more women with prior SGA (p = 0.002), BMI ≥ 30 kg/m^2^ (p<0.001), chronic hypertension (p<0.001), prior PE (p<0.001) and prior gestational diabetes (p = 0.041).

**Table 2:** Baseline characteristics of study groups.

Characteristics	LRW N = 51	HRW N = 49	HRPE N = 42	PEAGA n = 33	PESGA n = 9
Age, years^a^	29 (5)	33 (6)	31(6)	31.0(6.5)	31.0(8.0)
BMI, pre-pregnancy, kg/m^2^ ^b^	22.6 (2.4)	28.5 (9.7)	29.3(10.6)	29.6(11.0)	29.0(14.8)
Primiparous, n (%)^c^	30 (59)	12 (25)	10(24)	6(18.20)	4(44.4)
Infertility treatment, n (%)	4 (9)	7 (15)	5(14)	4(14.8)	1(11.1)
Chronic disease, n (%)^c^	10 (17)	27 (46)	22(37)	17(51.5)	5(55.6)
BMI ≥ 30 kg/m^2^ ^c^	1 (3)	21(43)	16(38)	13(38.2)	3(37.5)
Prior pre-eclampsia, n (%)^c^	0	14(29)	21(50)	19(57.6)	2(22.2)
SGA in previous pregnancy, n (%)^d^	0	9(18)	4(10)	2(6.1)	2(22.2)
Chronic hypertension^c^	0	14 (29)	10(24)	8(24.2)	2(22.2)
Prior GDM, n (%)^e^	0	5 (10)	4(10)	4(11.8)	0(0.0)
Type 1 DM, n (%)^f^	0(0.0)	1(2.0)	0(0.0)	0(0.0)	0(0.0)
Prior fetus mortus, n (%)	0	2(4)	1(2)	1(2.9)	0(0.0)
SLE, n (%)	0(0.0)	0(0.0)	0(0.0)	0(0.0)	0(0.0)
Sjögren´s syndrome, n (%)	0(0.0)	1(2.0)	0(0.0)	0(0.0)	0(0.0)

**a** Difference across the groups p < 0.001, no difference across the high-risk groups, median and IQR presented

**b** Difference between LRW vs. HRW or HRPE, p<0.001 for both comparisons, median and IQR presented

**c** Difference across the groups (p<0.001), no difference across the high-risk groups

**d** Difference across the groups (p = 0.002), no difference across the high-risk groups

**e** Difference across the groups **(**p = 0.041), no difference across the high-risk groups

**LRW** = low-risk women, **HRW** = high-risk women, **HRPE** = high-risk women who developed pre-eclampsia, **PEAGA** = pre-eclamptic women with appropriate for gestational age fetuses, **PESGA** = pre-eclamptic women with small for gestational age fetuses, IQR = interquartile range, BMI = body mass index, SGA = small for gestational age, GDM = gestational diabetes, DM = diabetes mellitus, SLE = systemic lupus erythematosus

### Pregnancy characteristics

The pregnancy characteristics of LRW, HRW and HRPE and the subgroups of SGAPE and AGAPE are shown in Table 3. LRW gained more weight during pregnancy compared to HRW and HRPE (p = 0.009 and p = 0.028, respectively) and the mean uterine artery pulsatility index in the first trimester was lower (p<0.001 for both comparisons). There was more gestational diabetes in HRW and HRPE compared to LRW (p = 0.014). Women in the HRPE group delivered earlier than in the HRW or LRW groups (p<0.001) and in the HRW group earlier than in the LRW group (p<0.001). There were more deliveries with vacuum extraction among HRW than HRPE and LRW (p = 0.006). Women in the HRPE group delivered smaller infants than in the HRW (p<0.001) or LRW (p = 0.015) groups. There was no difference in birth weight SD score between HRW and LRW (p = 0.146). Women in the PESGA group delivered earlier (median 31.9 GW) than in the other groups (p<0.001 in all pairwise comparisons).

**Table 3:** Pregnancy characteristics of study groups.

Pregnancy characteristics	LRW N = 51	HRW N = 49	HRPE N = 42	PEAGA n = 33	PESGA n = 9
Weight gain during pregnancy, kg**(%)**^a^	14.8(5.5)	11.7(9.0)	12.0(6.6)	12.3 (5.4)	11.1(10.4)
Gestational diabetes, n(%)^b^	4 (7.8)	13(26.5)	12(28.6)	10 (30.3)	2(22.2)
I trimester mean UTA PI	0.93(0.26)^c^	1.07(0.40)^d^	1.25(0.47)^e^	1.19(0.48)^f^	1.41(0.42)^g^
I trimester MAP, mmHg^h^	86.3(7.7)	97.0 (11.4)	101.6(13.3)	100.6 (13.0)	105.2(14.5)
Highest MAP, mmHg^i^	94.0(9.7)	105.0(12.7)	127.8(10.8)	128.3(16.7)	128.7(14.5)
Highest proteinuria, g/day^j^	0	0	1.5(2.3)	1.06(2.18)	2.30(5.20)
Gestational weeks at birth^k^	40.6(1.3)	39.9 (2.1)	38.4(3.2)	38.7(2.4)	31.9(5.6)
Mode of delivery, n (%)					
Vaginal	33 (65.0)	34 (69.4)	26 (61.9)	20 (61.0)	6 (66.7)
Vacuum extraction^k^	8 (16.0)	0 (0.0)	6 (14.3)	5 (15.2)	1 (11.1)
Elective cesarean section	1 (2.0)	3 (6.1)	1 (2.4)	0 (0.0)	1 (11.1)
Cesarean section during labor	8 (16.0)	12 (24.5)	8 (19.0)	7 (21.2)	1 (11.1)
Umbilical artery pH^l^	7.24 (0.09)	7.27(0.08)	7.27(0.08)	7.24(0.09)	7.22(0.11)
Newborn birthweight, g^m^	3524 (582)	3690(712)	3109(1259)	3370(687)	1358(1125)
Newborn birthweight, SD score^n^	-0.11	0.38	-0.78	-0.30	-2.51
Placental weight, g^o^	594(111)	641(128)	537(158)	582(143)	367(71)

**LRW** = low-risk women, **HRW** = high-risk women, **HRPE** = high-risk women who developed pre-eclampsia, **PEAGA** = women, who developed pre-eclampsia and had appropriate for gestational age fetuses, **PESGA** = women, who developed pre-eclampsia and had small for gestational age fetuses, UTA PI = uterine artery pulsatility index, MAP = mean arterial pressure, IQR = interquartile range, SD = standard deviation

**a** Difference across the groups (p = 0.015), no difference across the high-risk groups, median and IQR presented

**b** Difference across the groups (p = 0.025), no difference across the high-risk groups

**c** Differed from **d** and **e,** p<0.001 and from **f** (p = 0.006) and **g** (p = <0.001), **f** differed from **g** (p = 0.018), median and IQR presented

**h** Difference across the groups (p<0.001), no difference across the high-risk groups, mean and SD presented

**i** Difference between the groups (p<0.001) except between AGAPE vs. SGAPE, median and IQR presented

**j** No difference between AGAPE with SGAPE, median and IQR presented

**k** high-risk controls differed from low-risk women and high-risk women with subsequent PE, p = 0.006

**l** Difference between the groups (the highest p = 0.018) except between LRW vs. HRW, median and IQR presented

**m** No difference between the groups, mean and SD presented

**n** HRPE differed from HRW and LRW, p<0.001 and p = 0.003, respectively. SGAPE differed from LRW, HRW and AGASGA (all p-values <0.001) and AGAPE differed from HRW (p = 0.018), median and IQR presented

**o** HRPE differed from LRW and HRW (p = 0.015 and p<0.001, respectively), SGAPE differed from other groups (p<0.001), mean presented

**p** Difference between HRW and HRPE ((p = 0.001), SGAPE differed from other groups (p<0.001), mean and SD presented

### Outcomes

PE phenotypes are shown in Table 4. Of 42 women who developed PE, 33 (79%) gave birth to an AGA infant and 9 (21%) to an SGA infant. There were 21 women with severe and non-severe PE, respectively. Eleven women (26%) had early-onset PE and 32 (78%) late-onset PE.

**Table 4:** Distribution of pre-eclampsia phenotypes.

Women with SGA fetus	n	Women with AGA fetus	n
Severe EOPE	7	Severe EOPE	3
Non-severe EOPE	0	Non-severe EOPE	1
Severe LOPE	1	Severe LOPE	10
Non-severe LOPE	1	Non-severe LOPE	19
Total	9		33

SGA = small for gestational age, AGA = appropriate for gestational age, n = number of women, EOPE = early-onset pre-eclampsia, LOPE = late-onset pre-eclampsia

### Correlation and covariates

The Hpx and A1M concentrations of LRW at 12–14 GW correlated positively, after logarithmic normalization of the data, (Pearson r = 0.33, p = 0.022), as did they in the HRW group at 18–20 GW (Pearson r = 0.30, p = 0.039). Hpx and A1M concentrations did not correlate in other respects. The BMI was a significant covariate for the Hpx (main effect BMI, p = 0.001 in the RMANOVA model, Pearson r = 0.20, p<0.001), but not for the A1M concentration. Therefore, the Hpx concentration was adjusted for BMI. Also other covariates were tested: Hpx and A1M concentrations were adjusted for maternal age, ASA usage, gestational diabetes, MAP in the first trimester and mean uterine artery pulsatility index. None of the tested variables turned out to be a significant covariate for Hpx or for A1M.

### Hemopexin and alpha-1-microglobulin concentrations

At first trimester sampling the mean GW was 13.0 (11.9–15.9), at second sampling 19.4 GW (18.0–21.3) and at third sampling 27.1 GW (25.0–28.7). The geometric mean plasma concentrations of Hpx, Hpx adjusted for BMI and A1M are shown in Tables 5A, 5B and 6, respectively.

**Table 5:** Geometric mean plasma hemopexin concentrations (mg/ml) by subgroups at three different sampling points during pregnancy. A before adjustment for body mass index, B after adjustment for body mass index.

**Table 5. A**									
**Group**	**12–14 GW**	95% CI		**18–20 GW**	95% CI		26–28* **GW**	95% CI	
	**Hpx mg/ml**	Lower Bound	Upper Bound	**Hpx mg/ml**	Lower Bound	Upper Bound	**Hpx mg/ml**	Lower Bound	Upper Bound
**LRW N = 51**	1.20	1.13	1.28	1.19	1.12	1.26	1.03^e^	0.97	1.09
**HRW N = 49**	1.12^a^	1.06	1.19	1.11^b^	1.05	1.18	1.13	1.06	1.20
**HRPE N = 42**	1.27	1.19	1.36	1.25	1.17	1.33	1.20^f^	1.12	1.29
**PESGA n = 9**	1.29	1.11	1.49	1.17	1.02	1.34	1.12	0.97	1.31
**PEAGA n = 33**	1.27	1.18	1.36	1.27^c^	1.19	1.36	1.22^g^	1.13	1.32
**EOPE n = 11**	1.23	1.08	1.40	1.22	1.08	1.38	1.17	1.02	1.35
**LOPE n = 31**	1.28^a^	1.19	1.39	1.26^d^	1.18	1.36	1.21^h^	1.12	1.31
**SEV n = 21**	1.28	1.17	1.41	1.26	1.16	1.38	1.18	1.07	1.31
**MILD n = 21**	1.26	1.15	1.38	1.24	1.13	1.35	1.22^i^	1.11	1.35
**Table 5. B**									
**Group**	**12–14 GW**	95% CI		**18–20 GW**	95% CI		26–28* **GW**	95% CI	
	**Hpx_A mg/ml**	Lower Bound	Upper Bound	**Hpx_A mg/ml**	Lower Bound	Upper Bound	**Hpx_A mg/ml**	Lower Bound	Upper Bound
**LRW N = 51**	1.24	1.17	1.32	1.23	1.17	1.31	1.05	0.99	1.12
**HRW N = 49**	1.11	1.04	1.17	1.10	1.03	1.16	1.11	1.05	1.18
**HRPE N = 42**	1.24	1.13	1.31	1.23	1.15	1.31	1.18	1.10	1.26
**PESGA n = 9**	1.26	1.09	1.47	1.15	1.00	1.31	1.10	0.95	1.28
**PEAGA n = 33**	1.24	1.14	1.33	1.26	1.17	1.34	1.20	1.11	1.30
**EOPE n = 11**	1.21	1.06	1.38	1.19	1.06	1.35	1.15	1.00	1.33
**LOPE n = 31**	1.25	1.16	1.36	1.24	1.16	1.34	1.19	1.09	1.29
**SEV n = 21**	1.28	1.16	1.40	1.26	1.15	1.37	1.17	1.07	1.30
**MILD n = 21**	1.20	1.09	1.33	1.21	1.10	1.32	1.17	1.06	1.30

* Published earlier as medians [34].

GW = week of gestation, CI = confidence interval, Hpx = mean hemopexin concentration, A_Hpx = mean hemopexin concentration after adjustment for BMI, **LRW** = low-risk women, controls **HRW** = high-risk women, controls, **HRPE** = high-risk women who developed pre-eclampsia, **EOPE** = women who developed early-onset pre-eclampsia, **LOPE** = women, who developed late-onset pre-eclampsia, **SEV** = women, who had severe pre-eclampsia, **MILD** = women, who had non-severe (mild) pre-eclampsia, **PESGA** = women who developed pre-eclampsia and gave birth to an small for gestational age infant, **PEAGA** = women who developed pre-eclampsia and gave birth to an appropriate for gestational age infant

**a** p = 0.034 **b** lower than **c** p = 0.020 and **d** p = 0.041, **e** lower than **f** = 0.003, **g** p = 0.004, **h** p = 0.011 and **i** p = 0.029

**Table 6:** Geometric mean plasma alpha-1-microglobulin concentration (μg/ml) by subgroups at three different sampling points during pregnancy.

Group	12–14 GW	95% CI		18–20 GW	95% CI		26–28* GW	95% CI	
	A1M μg/ml	Lower Bound	Upper Bound	A1M μg/ml	Lower Bound	Upper Bound	A1M μg/ml	Lower Bound	Upper Bound
**LRW N = 51**	12.0^a^	11.0	13.0	11.9^g^	11.0	12.9	12.7^m^	11.6	13.7
**HRW N = 49**	12.9^b^	11.8	14.0	14.6^h^	13.5	15.9	15.2^n^	14.0	16.5
**HRPE N = 42**	15.6^c^	14.2	17.0	12.7^i^	11.7	13.9	13.7	12.5	15.0
**PESGA n = 9**	15.5	12.7	19.0	15.4	12.8	18.6	17.6^o^	14.5	21.3
**PEAGA n = 33**	15.5^d^	14.0	17.1	12.1^j^	11.0	13.3	12.8	11.6	14.1
**EOPE n = 11**	15.9	13.3	19.0	14.6	12.3	17.4	16.1	13.5	19.2
**LOPE n = 31**	15.4^e^	13.9	17.1	12.1^k^	11.0	13.4	13.0	11.7	14.4
**SEV n = 21**	16.6^f^	14.6	18.9	13.7	12.1	15.5	15.1	13.3	17.2
**MILD n = 21**	14.6	12.8	16.5	11.8^l^	10.5	13.4	12.4^p^	10.9	14.1

* Published earlier as medians [34].

GW = weeks of gestation, CI = confidence interval, A1M = mean alpha-1-microglobulin concentration, **LRW** = low-risk women, **HRW** = high-risk women, **HRPE** = high-risk women who developed pre-eclampsia, **PESGA** = women who developed pre-eclampsia and gave birth to an small for gestational age infant, **PEAGA** = women who developed pre-eclampsia and gave birth to an appropriate for gestational age infant, **EOPE** = women who developed early-onset pre-eclampsia, **LOPE** = women, who developed late-onset pre-eclampsia, **SEV** = women, who had severe pre-eclampsia, **MILD** = women, who had non-severe (mild) pre-eclampsia

**a** vs. **b** p = 0.017, **a** vs. **c** p<0.001, **a** vs. **d** p = 0.017, **a** vs. **e** p = 0.002, **a** vs. **f** p = 0.001, **b** vs. **d** p = 0.036, **b** vs. **f** p = 0.017, **g** vs. **h** p = 0.001, **h** vs. **i** p = 0.038, h vs. **j** p = 0.020, **h** vs. **k** p = 0.028, **h** vs. **l** p = 0.026, **m** vs. **n** p = 0.005, **m** vs. **o** p = 0.032, **n** vs. **p** p = 0.048

### Hemopexin

#### All pre-eclampsia vs. controls

**Fig 2:**
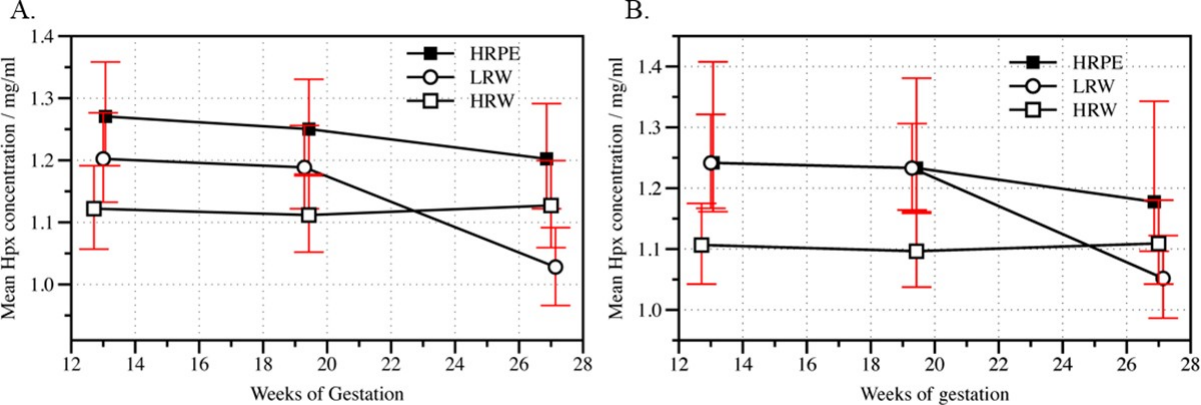
Geometric mean of plasma hemopexin concentration in three study groups by duration of gestation. The geometric means and the 95% confidence intervals (red bars) of the plasma hemopexin (Hpx) concentration among low-risk women (LRW) and high-risk women who did (HRPE) and did not (HRW) develop pre-eclampsia. Data shown by weeks of gestation **(A)** before and **(B)** after adjustment for body mass index.

There was a difference in the plasma Hpx concentration and the change of the Hpx concentration between the three study groups, main effect Group, p = 0.002 and interaction effect Time x group, p = 0.021 (Table 5A, Fig 2A), and the result remained significant after adjustment for BMI, main effect Group, p = 0.004 and interaction effect Time x group, p = 0.010 (Table 5B and Fig 2B). When adjusted for BMI, the plasma concentration of hemopexin in the LRW and HRPE groups changed similarly from 12–14 to 26–28 weeks of gestation (main effect Group, p = 0.374, interaction effect Time x group, p = 0.063) while the Hpx concentration of HRW was at the same level throughout the study period and therefore differed from the Hpx concentration of LRW and HRPE (interaction effect Time x group, p = 0.006, Fig 2B).

The level of plasma Hpx in the HRW group during the first half of the pregnancy (from 12–14 to 18–20 GW) was lower than in the LRW and HRPE groups (main effect Group, p = 0.001, Table 5A, Fig 2A). This difference persisted after adjustment for BMI (main effect Group, p<0.001, Table 5B, Fig 2B).

After midgestation, the mean Hpx concentration decreased in the LRW group (-0.16 mg/ml) and increased slightly in the HRW group (0.01 mg/ml). These changes differed significantly from each other (p = 0.012) over time (between 18–20 and 26–28 GW). During the same period, the mean Hpx concentrations were higher in the HRPE than the LRW group (main effect Group, p = 0.015, changes in Hpx concentration -0.05 vs. -0.16, respectively, p = 0.056) and the HRW group (main effect Group, p = 0.039). The changes in Hpx concentrations did not differ between HRPE and HRW after midgestation (-0.05 vs. 0.01, respectively, p = 1.000, Table 5A, Fig 2A). After adjustment for BMI there was no difference in the Hpx concentration between LRC and HRPE.

#### Small for gestational age and appropriate for gestational age (Table 5)

The mean plasma Hpx concentration in the PESGA group decreased over time from 12–14 to 26–28 GW, but in the PEAGA group it remained at the same level from 12–14 to 18–20 GW and then decreased from 18–20 to 26–28 GW. These differences between PESGA and PEAGA were not statistically significant (main effect Group, p = 0.449, interaction effect Time x group, p = 0.449).

When analyzed separately at each sampling point, the Hpx concentration in the PEAGA group was higher than in the HRW group (1.27 vs. 1.11 mg/ml, respectively, p = 0.020) at 18–20 GW. At 26–28 GW PEAGA had a higher Hpx concentration than LRW (1.22 vs. 1.03 mg/ml, respectively, p = 0.004).

#### Early-onset and late-onset pre-eclampsia (Table 5)

Although the Hpx concentrations of high-risk women who developed late-onset PE compared to high-risk women who developed early-onset PE were constantly higher at each sampling point, the difference was significant neither before (main effect Group p = 0.575 and interaction effect Time x group p = 0.998) nor after adjustment for BMI (p = 0.574 and p = 0.996, respectively).

When analyzed separately at each sampling point, the Hpx concentrations of women who developed late-onset PE was higher than in HRW at 12–14 GW (1.28 vs. 1.12 mg/ml, respectively, p = 0.034) and at 18–20 GW (1.26 vs. 1.11 mg/ml, respectively, p = 0.041). The Hpx concentrations of women who developed late-onset PE was higher than in the LRW group at 26–28 GW (1.21 vs. 1.03, respectively, p = 0.011).

#### Severe and non-severe pre-eclampsia (Table 5)

There was no difference in plasma Hpx concentrations between high-risk women with severe PE and high-risk women with non-severe PE neither before (main effect Group p = 0.933, interaction effect Time x group p = 0.726) nor after adjustment for BMI (main effect Group p = 0.378 and interaction effect Time x group p = 0.759). When analyzed separately at each sampling point, the Hpx concentrations of women who developed non-severe PE were higher than in LRW at 26–28 GW (1.22 vs. 1.03 mg/ml, respectively, p = 0.029).

### Alpha-1-microglobulin

#### All pre-eclampsia vs. controls

**Fig 3:**
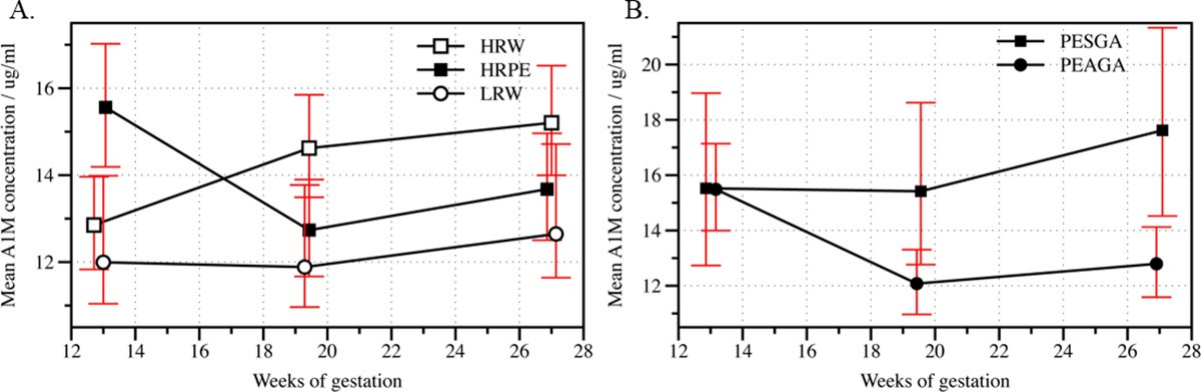
Geometric mean of alpha-1-microglobulin concentration in three study groups and in pre-eclamptic women with small for gestational age and appropriate for gestational age fetus by weeks of gestation. The geometric means of the plasma alpha-1-microglobulin (A1M) concentration by weeks of gestation (**A)** among low-risk women (LRW) and high-risk women, who developed pre-eclampsia (HRPE) and among high-risk women who did not develop pre-eclampsia (HRW) and **(B)** among women who developed pre-eclampsia and gave birth to a small for gestational age infant (PESGA) and to an appropriate for gestational age infant (PEAGA). Red bars indicate 95% confidence intervals.

There was a difference in A1M concentrations and in the changes of the concentrations longitudinally between HRPE, HRW and LRW (main effect Group, p = 0.009 and interaction effect Time x group, p<0.001) (Fig 3A and Table 6). In further analyses of A1M, the concentrations of A1M in the HRPE and HRW was higher than in the LRW group from 12–14 to 18–20 GW (Fig 3A, Table 6, main effect Group, p = 0.021). The mean concentration of A1M in the HRPE decreased more than in the LRW (-2.8 vs. -0.1 μg/ml, interaction effect Time x group, p<0.001) from 12–14 to 18–20 GW. While the A1M concentration in the HRPE decreased, the A1M concentration in HRW increased (-2.8 vs. 1.8 μg/ml, respectively, interaction effect Time x group, p<0.001) between the first sampling at 12–14 GW and the second sampling at 18–20 GW. The change in A1M in the HRW and LRW groups between 12–14 and 18–20 GW differed also (1.8 vs. -0.1μg/ml, interaction effect Time x group, p<0.001). The A1M concentration in the HRW group was higher compared to the LRW and HRPE groups between the second sampling point at 18–20 GW and the third sampling point at 26–28 GW (main effect Group, p = 0.002).

#### Small for gestational age and appropriate for gestational age (Table 6)

The mean plasma A1M concentration in the PEAGA group decreased from 12–14 to 18–20 GW compared to PESGA, but the change was not statistically significant (-3.4 μg/ml vs. -0.1 μg/ml interaction effect Time x group, p = 0.011, after Bonferroni correction p = 0.066). From midgestation onwards (from 18–20 to 26–28 GW) the A1M concentration was higher in the PESGA group compared to the PEAGA group (main effect Group, p = 0.008, after Bonferroni correction p = 0.048) (Fig 3B).

#### Early-onset and late-onset pre-eclampsia (Table 6)

The mean plasma A1M concentration among high-risk women who developed early-onset PE was constantly higher at each sampling point than among high-risk women who developed late-onset PE. The difference was not significant (main effect Group p = 0.134 and interaction effect Time x group p = 0.082).

#### Severe and non-severe pre-eclampsia (Table 6)

The mean plasma A1M concentration was higher at each of the three sampling points among high-risk women who developed severe PE compared to high-risk women who developed non-severe PE. However, the difference was not significant (main effect Group p = 0.055, interaction effect Time x group p = 0.668).

### Effects of LDA treatment on biomarker concentrations

Ten women had LDA treatment from the first trimester until 35 GW. Three of these women were in the HRW, seven in the HRPE group. Although women with LDA treatment were unevenly distributed between the study groups (p = 0.022), the mean Hpx and A1M concentrations did not differ between women who did and did not have LDA treatment (S1 Table). LDA usage was not a significant covariate in RMANOVA for Hpx (p = 0.196) or for A1M (p = 0.312).

## Discussion

To our knowledge, this is the first study to investigate plasma Hpx and A1M concentrations over the duration of pregnancy among women at high risk for PE (some of whom ultimately developed PE) and among women at low risk for PE.

Since there are no studies on longitudinal plasma Hpx values during pregnancy, the natural variations of plasma Hpx concentration over the duration of normal pregnancy are not known. We hypothesized that the longitudinal Hpx concentration would be lower in HRPE compared to the other groups due to a PE-induced increase in HbF levels in the maternal circulation and by subsequent activation of the Hb scavenger system, which in turn would cause Hpx depletion. However, the plasma Hpx concentration among the HRPE did not differ from the plasma Hpx concentration among the LRW and it was significantly higher compared to the HRW during the first half of pregnancy, suggesting a protective role for low Hpx concentration in HRW. There is some contradiction between our study results and those of Anderson et al. who found that plasma collected in the first trimester had lower Hpx concentrations among women who subsequently developed early-onset PE compared to women who did not develop PE [19]. The discrepancy is due to the multifactorial mechanisms affecting the concentration and activity of Hpx. Changes in the plasma hemopexin concentration reflect the balance between, on the one hand, hepatic synthesis of Hpx and, on the other, depletion of Hpx due to heme scavenging.

It is also of note that Hpx is an acute-phase plasma protein and its expression and activity may be modulated by conditions associated with systemic inflammations and oxidative stress mediated by ROS. The expression of Hpx can be induced through a ROS-dependent mechanism in type 1 diabetes mellitus [38]. In the same way, the expression of Hpx might be induced in PE during pregnancy due to ROS-mediated oxidative stress, which would explain the increased level of hemopexin in PE as shown in the present study, where the obesity, diabetes mellitus and chronic hypertension were among the inclusion criteria of women with high-risk pregnancies. These conditions are known to induce oxidative stress mediated by ROS and might predispose to increased plasma Hpx concentrations [39–41].

Some of the differences in mean Hpx concentration between the study groups could be explained by the effect of BMI, although most of the differences remained significant after adjustment of the data for BMI. Our hypothesis was that the BMI-adjusted Hpx concentrations might reflect the role of the placenta in the pathophysiology of PE more reliably. It is, however, also known that obesity itself is an independent risk factor for PE [42] and therefore it is relevant to present both unadjusted and adjusted results when longitudinal changes in plasma Hpx concentrations are presented.

In the present study, the plasma Hpx concentration started to decrease from the first trimester in PESGA, as we had hypothesized (Table 5A and 5B). The concentration of cell-free HbF is higher in the feto-placental circulation in FGR than in normal pregnancies [22]. This being the case cell-free HbF could very well leak into the maternal circulation, injuring the placental barrier and leading to Hpx depletion. However, the number of women in this group was rather small and the difference in Hpx and in the changes in the Hpx concentration were not statistically significant (Table 5A and 5B). The risk of type II error in this situation is possible, since the Hpx concentration clearly developed as hypothesized, but the sample size was small.

The mean plasma A1M concentration was higher in the HRPE than the HRW and LRW in the first trimester. The present study confirms previous findings [17, 19] (Table 6, Fig 3A) and contributes additional evidence that suggests differences in A1M changes among pre-eclamptic women, depending on fetal growth (Table 6, Fig 3B). We found that PE women with SGA fetuses had consistently higher plasma level of A1M than women in the HRW and LRW, presumably as a response to increased fetal and maternal cell-free HbF concentrations throughout the duration of pregnancy. It is tempting to hypothesize that, in cases of PE complicated by SGA, cell-free HbF, originally present in the feto-placental circulation, leaks into the maternal circulation, leading to activation of endogenous Hb- and heme-scavenging systems, of which A1M is an important component. However, cell-free HbF may not be the sole factor affecting the plasma level of A1M since its synthesis is induced by both cell-free HbF and by ROS [43]. The increased plasma A1M level in the HRW group compared to the LRW group might be explained as the result of activation of A1M following systemic inflammation and oxidative stress not related to cell-free HbF. This is speculative since the present study did not study the plasma cell-free HbF level.

One unexpected finding was that the average plasma level of A1M was lower in the PEAGA group at midgestation than in the PESGA group. This may be explained by the fact that the timing of placental dysfunction differs between PESGA and PEAGA. In the latter case, PE develops later during gestation. According to the revised two-stage model of PE, late-onset PE develops when the maturing placenta outgrows the capacity of the uterus in late pregnancy, resulting in restricted intervillous perfusion, syncytiotrophoblast stress and release of several inflammatory factors into the maternal circulation, including cell-free HbF [44]. It can thus be anticipated that an increase in the plasma A1M level occurs at later stages of gestation in PEAGA than in PESGA. In the present study, the plasma A1M level was not evaluated after 28 weeks of gestation, and this leaves this hypothesis unanswered at present. However, the different plasma Hpx and A1M profiles in PESGA compared to PEAGA (Table 5 and Table 6) may reflect earlier and more profound damage of the placenta-blood barrier in PESGA. Hpx is consumed to a higher degree in FGR due to its scavenging property and not as much induced by ROS as A1M is. While A1M also has scavenging properties, it is induced to a higher degree by ROS and is more active against ROS than Hpx.

### Strength, limitations and recommendations

The strength of the study is a carefully described study cohort, where both women with predetermined risk factors for PE and a low-risk reference group were prospectively recruited and studied.

A limitation of our study is that ten high-risk women had LDA treatment from the first trimester onward until 35^+0^ GW. These women were unevenly distributed throughout the study groups. However, LDA use was not a significant covariate for Hpx or A1M concentrations in RMANOVA.

Due to the case-control set-up, this is a preliminary study and results should be validated in another cohort.

## Conclusions

The present study confirms the results of recent studies that the plasma concentration of A1M is higher in HRPE compared to HRW and to LRW in the first trimester. We also found that the concentration of A1M in PESGA remains high during the first half of pregnancy compared, but in PEAGA it decreases. In women who develop PE, high A1M concentration at and after midgestation is associated with SGA.

HRW have different plasma Hpx and A1M concentrations over time during pregnancy compared to HRPE and LRW. This phenomenon may be associated with a reduced risk of PE despite clinical risk factors. Risk factors related to induction of oxidative stress, such as diabetes mellitus, obesity and chronic hypertension, and fetal growth restriction, appeared to be significant in this respect. Future risk evaluation should measure the balance between oxidative stress markers in relation to scavenger proteins such as Hpx and A1M.

## Supporting information

S1 Table Effects of low-dose acetylsalicylic acid (100mg/d) treatment on biomarker concentrations. * ANOVA adjusted for group (HRW or HRPE). Hpx = geometric mean of plasma hemopexin concentration, A1M = geometric mean of plasma alpha-1-microglobulin concentration, LDA = low-dose acetylsalicylic acid (100mg/d).
(DOCX)
