# Supplementary material for: Longitudinal changes in plasma hemopexin and alpha-1-microglobulin concentrations in women with and without clinical risk factors for pre-eclampsia
Source: PLoS One. 2019 Dec 16;14(12):e0226520. doi: 10.1371/journal.pone.0226520 (PMC6913989; doi:10.1371/journal.pone.0226520)
Supplement: S1 Table — * ANOVA adjusted for group (HRW or HRPE). Hpx = geometric mean of plasma hemopexin concentration, A1M = geometric mean of plasma alpha-1-microglobulin concentration, LDA = low-dose acetylsalicylic acid (100mg/d). (DOCX) [file pone.0226520.s001.docx]

| **12-14 GW** | **Hpx mg/ml** | p-value* | **A1M µg/ml** | p-value* |
| --- | --- | --- | --- | --- |
| No LDA, n=80 | 1.18 | 0.074 | 14.3 | 0.060 |
| LDA, n=9 | 1.35 |  | 11.8 |  |
| **18-20 GW** |  |  |  |  |
| No LDA, n=79 | 1.19 | 0.139 | 13.7 | 0.127 |
| LDA, n=9 | 1.07 |  | 11.7 |  |
| **26-28 GW** |  |  |  |  |
| No LDA, n=78 | 1.14 | 0.057 | 14.6 | 0.517 |
| LDA, n=9 | 1.32 |  | 13.6 |  |
